# Urography and CT Features of Primary Small Cell Carcinoma of the Ureter: A Case Report

**DOI:** 10.5812/iranjradiol.4779

**Published:** 2013-08-30

**Authors:** Jianfeng Yang, Zhenhua Zhao, Jiayin Ni, Wenpin Dong, Ning Wang, Boyin Wang

**Affiliations:** 1 Department of Radiology, Shaoxing People's Hospital, Zhejiang University, Shaoxing, Zhejiang, China; 2 Department of Urology, Shaoxing People's Hospital, Zhejiang University, Shaoxing, Zhejiang, China; 3 Department of Pathology, Shaoxing People's Hospital, Zhejiang University, Shaoxing, Zhejiang, China

**Keywords:** Ureter, Carcinoma, Small Cell, Urography, Tomography, X-Ray Computed

## Abstract

Primary small cell carcinoma of the ureter is an extremely rare disease, only several cases have been reported worldwide so far. We report a 70-year-old woman who was examined with intravenous urography and abdominal computed tomography and was diagnosed as small cell carcinoma confirmed by pathology. We describe and discuss the urography and computed tomography findings of this case.

## 1. Introduction

Small cell carcinoma (SCC) is usually found in the lungs, but its extrapulmonary counterpart is rarely encountered (1). According to Remick, the incidence of extrapulmonary SCC is 0.1% to 0.4% (2). SCC of the genitourinary tract has been reported previously in the urinary bladder. Primary small cell carcinoma (PSCC) of the ureter is a rare entity; only several cases have been reported worldwide (3). The cause of SCC of the ureter may be related to smoking, but it remains unclear (4). Previous reports have discussed the clinical characteristics and therapeutic methods for SCC (3-5). To date, no report has discussed urography and computed tomography (CT) findings of PSCC of the ureter in detail; thus, we report the urography and CT findings of this PSCC of the ureter.

## 2. Case Presentation

A 70-year-old woman presented with left-sided flank pain without symptoms of bladder irritation and gross hematuria. The pain was accompanied by nausea. The patient had no smoking history. The ultrasonography revealed a 6mm diameter calculus in the distal region of the left ureter with hydronephrosis. Then she was examined using intravenous urography (IVU), abdominopelvic CT, and chest radiography.

**Figure 1.**
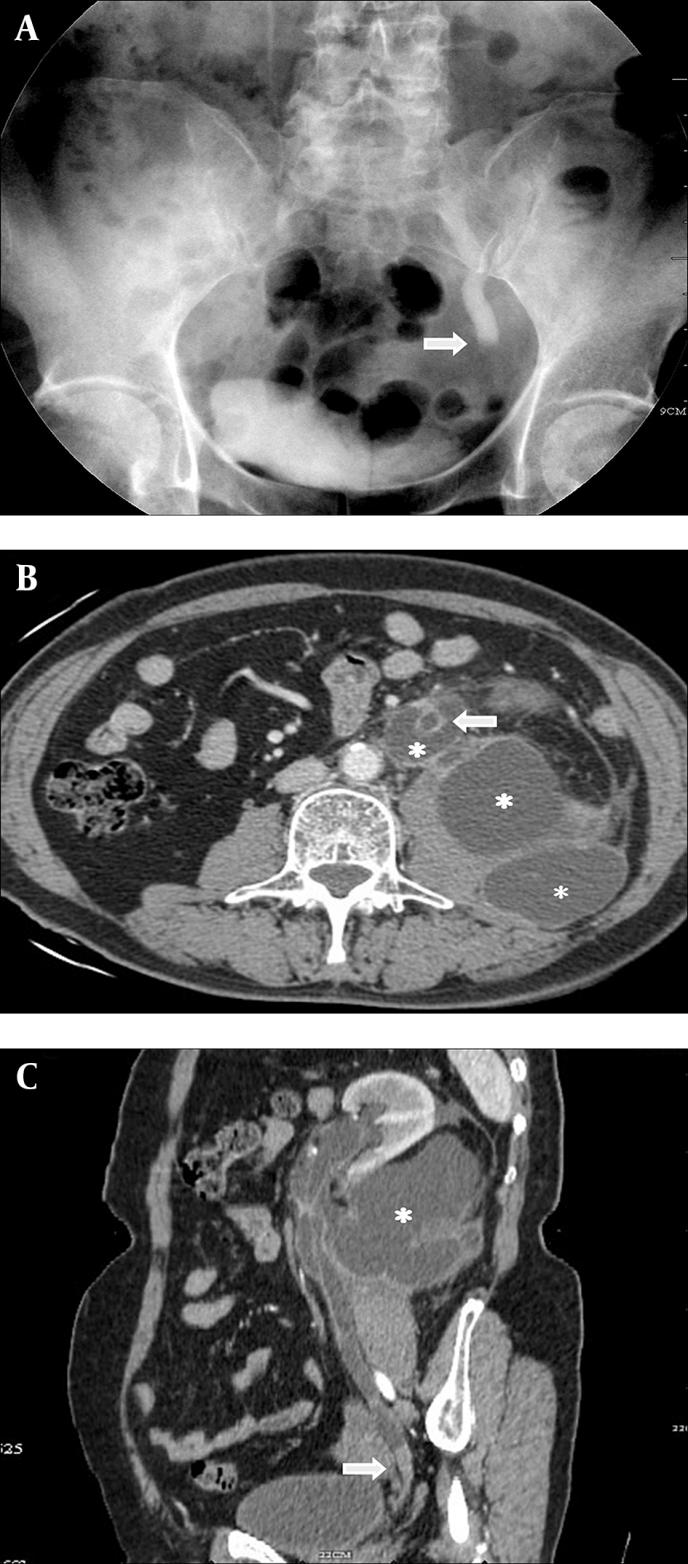
A, The IVU shows the dilated ureter with amputated end (arrow). B, Computed tomography of the abdomen indicates pronounced hydroperinephrosis (asterisk) and dilated ureter (arrow). C, The sagittal oblique reconstruction of left ureter shows a lesion at the end of ureter (arrow) and hydroperinephrosis (asterisk).

The IVU showed the amputated end of the left ureter with hydroureterosis (Figure 1 A). CT indicated pronounced left hydroperinephrosis and thickening in the perirenal tissues and left upper ureteral wall (Figure 1 B). A mass sized 9.8×11.8 mm was seen that exhibited a slight hyperattenuation. The mass was mostly homogeneous, but several small spots of hypoattenuation were detected in the lesion. Attenuation of the lesion was 62.5 Hounsfield units (HU) on the corticomedullary phase; it reached 92 HU on the nephrographic phase. Abdominal multiplanar reconstruction (MPR) showed the mass near the end of the ureter and a thickened, irregular wall of the upper ureter with pronounced hydroperinephrosis was seen (Figure 1 C). We did not observe lymphadenopathy near the pelvis, retroperitoneum and para-aorta. No lesion was detected in the chest radiograph. 

**Figure 2.**
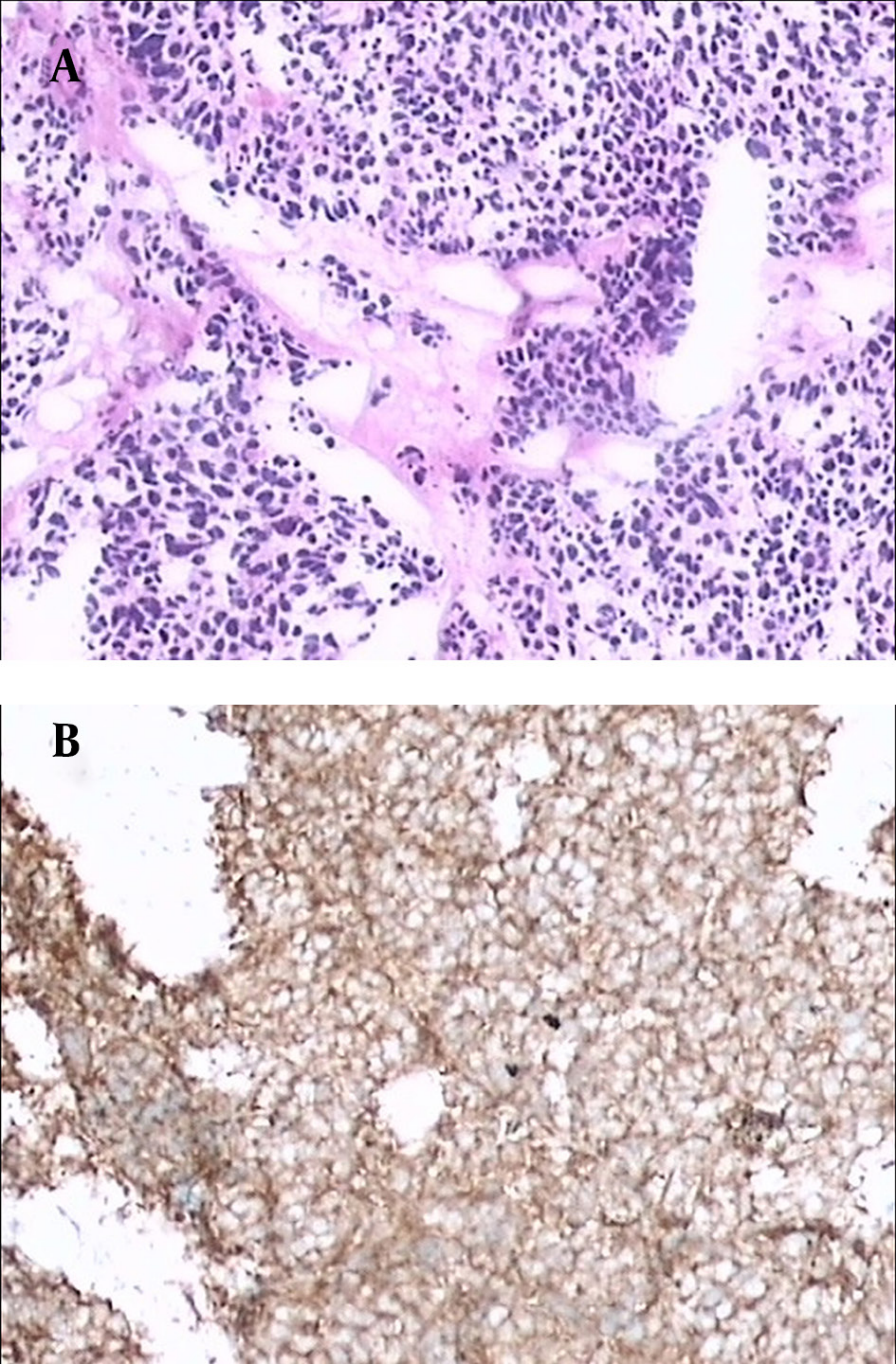
A, Cancer cells exhibit hyperchromatic nuclei, round to fusiform shape, and high mitotic activity with little cytoplasm and an absence of nucleoli (H&E). B, The immunohistochemical staining of the specimen is positive for CgA.

The patient underwent left nephroureterectomy. Macroscopically, a 1.6×1.2×0.5 cm grayish white lesion was identified between the bladder and end 3.5 cm of the ureteral specimen. Histological and immunohistochemical staining examination confirmed PSCC of the ureter (Figure 2). The abdominal and pelvic CT and chest radiography that was performed 9 months after the operation indicated massive metastasis in the liver and lung, numerous lymphadenopathies in the retroperitoneum and para-aortic region and some implantation of metastatic cells in the bladder and left psoas major muscle. 

## 3. Discussion

There are two hypotheses regarding the histopathogenesis of urinary tract SCC. One indicates that it originates from intrinsic neuroendocrine cells within the normal genitourinary tract derived from the neural crest during embryogenesis (6). Whereas the other hypothesis suggests that it results from a transformation of pluripotent epithelial reserve cells in the genitourinary tract that exhibit the ability to generate any cell type (7). According to Kim et al. (4), SCC of the ureter combined with other components such as transitional cell carcinoma, adenocarcinoma, squamous cell carcinoma and carcinoma sarcomatodes, supports the second hypothesis. However, in this case, there are no other components, thus it is compatible with the first hypothesis.

Because of high mitotic activity (3, 8-10) and the high tumoral mass (11), SCC of the ureter usually obstructs the ureter completely. The IVU or the MPR CT shows the end of the contrast agent is amputated completely, and the ureter above the obstruction and renal pelvis exhibits severe dilatation. When the pressure in the ureter and renal pelvis is extremely high and accompanied with the injured ureteral wall by cancer cell infiltration, it may cause ureterostoma. The perirenal tissue is thickened with stimulation of urine, thus, the thickened perirenal tissue and hydroperinephrosis present as multiple cystic structure. Severe and long-standing obstruction can give rise to renal insufficiency or renal failure and the excretion of contrast medium would not be visualized in IVU or CT after contrast administration. In this patient, we do not find these changes. We presume that it is associated with these two reasons: the pressure in the left renal pelvis decreased after hydroperinephrosis, and compensation of the right renal function started.

In this case, the enhancement of the lesion increased equal to 29.5 HU from the corticomedullary phase to the nephrographic phase. It indicates PSCC of the ureter has slight-midrange enhancement. The extent of slight-midrange enhancement at CT reflects the underlying extent of nodular angiogenesis (12). The attenuation of the mass was mostly homogeneous, but several small spots of hypoattenuation also exhibited in the nodule, because the lesions outgrow their blood supply, resulting in necrosis.

The boundary between the mass and the ureteral wall could not be discerned. This characteristic may be due to the tumor mass infiltrating the ureteral wall completely, even encasing it leading to an early and extensive metastasis in PSCC. We did not find the metastasis in other organs prior to or during surgical operation. However, 9 months after the surgical procedure we found extensive metastases scattered over the liver and lung and numerous lymphadenopathies around the retroperitoneum and para-aorta, as well as metastatic implantation in the bladder. This PSCC of the ureter in particular impacts the ureterostoma and causes hydroperinephrosis; so metastasis to the psoas major also occurs.

It may be difficult to differentiate PSCC of the ureter from transitional cell carcinoma (TCC) that account for 5% to 7% of all urinary tract tumors (13). But there are also some differentiations between PSCC and TCC of the ureter on urography and CT. The appearance of TCC of the ureter on excretory urography is filling defect with or without proximal hydroureteronephrosis or the goblet sign that is thought to be caused by the slow growing tumor expanding the ureteral lumen (14). On CT, some TCC of the ureter manifest as ureteral wall thickening with luminal narrowing or a focal intraluminal mass; such as PSCC, some TCCs are soft tissue masses with homogeneous density; the mass may even infiltrate the ureteral wall (15). The mass usually exhibits mild enhancement. The lymph node metastases or distant metastases also occur in some TCC patients (16), but the incidence is far less than PSCC of the ureter. CT urography is a useful imaging method for assessing both PSCC and TCC, but the final diagnosis depends on histopathologic examination.

Limitations of our report include only one case of PSCC of the ureter and no plain and excretory phase of abdominal CT; nevertheless, PSCC of the ureter is such a rare disease that no report has described the IVU and CT appearances of PSCC of the ureter in detail. Diagnosis by cystoscopy may cause trauma of the ureteral wall and increase possibility of metastasis. So describing and discussing the urography and CT features of this PSCC may also aid in the clinical diagnosis and promote timely treatment for affected patients, however nephroureterectomy is the optimal method by which PSCC of the ureter is diagnosed and managed.
